# Association Between Diet-Related Inflammation and COPD: Findings From NHANES III

**DOI:** 10.3389/fnut.2021.732099

**Published:** 2021-10-18

**Authors:** Haiyue Liu, Xilan Tan, Zuheng Liu, Xiaobo Ma, Yanqing Zheng, Bo Zhu, Gangsen Zheng, Yuehong Hu, Lili Fang, Guolin Hong

**Affiliations:** ^1^The Department of Laboratory Medicine, The First Affiliated Hospital of Xiamen University, Xiamen, China; ^2^Xiamen Key Laboratory of Genetic Testing, Xiamen, China; ^3^Division of Infection Medicine, Zhujiang Hospital, Southern Medical University, Guangzhou, China; ^4^Department of Cardiology, The First Affiliated Hospital of Xiamen University, Xiamen, China

**Keywords:** chronic obstructive pulmonary disease, dietary inflammatory index, nutrition, National Health and Nutrition Examination Survey, diet

## Abstract

**Background and Aims:** Little is known about diet-related inflammation in chronic obstructive pulmonary disease (COPD). In this study, we aimed to explore the association between COPD and dietary inflammatory index (DII) scores in adults over 40 years old.

**Methods:** Data were obtained from the 2013 to 2018 National Health and Nutrition Examination Survey (NHANES). In the present study, 9,929 participants were included and analyzed. The DII score was calculated and divided into tertiles. Logistic regression analysis was performed to determine the odds ratios of DII tertiles.

**Results:** Participants were categorized into COPD (565, 5.69%) and non-COPD groups (9,364, 94.31%) according to interview information. COPD individuals had higher DII scores than non-COPD individuals (0.429 ± 1.809 vs. −0.191 ± 1.791, *p* < 0.001). The highest DII score tertile included 46.55% of COPD individuals was associated with lower family incomes and education and a higher smoking rate (*p* < 0.01). The odds ratios (95% CIs) of COPD according to logistic regression were 0.709 (0.512–0.982) for T1 and 0.645 (0.475–0.877) for T2 of the DII score (*p* = 0.011).

**Conclusion:** Higher DII scores were positively correlated with COPD in participants over 40 years old. These results further support that diet can be used as an intervention strategy for COPD management.

## Introduction

Chronic obstructive pulmonary disease (COPD) is an inflammatory disease that is characterized by irreversible and progressive airflow limitation and has become a major public health problem worldwide due to its high morbidity and mortality (1). A report estimated that the global prevalence of COPD (stage II or above) is ~10% (12% for men and 9% for women) (2). The prevalence of COPD in people aged ≥40 years (9.7%) was significantly higher than that in people younger than 40 years old (2.7%) (3). COPD is one of the most burdensome diseases according to the World Health Organization's burden of disease.

The risk factors for COPD are multifactorial, including smoking, diet, genetics and environment, and alterations in dietary patterns may play a role in COPD progression (4–6). Additionally, the role of diet in inflammatory lung diseases has been indicated in previous studies (7, 8). A western diet increases the risk of COPD (9), while a diet high in vegetables, fruit, and fish is associated with a lower risk (10). Dietary Inflammation Index (DII) is an effective tool for measuring the inflammatory potential of an individual's diet (11). A higher DII score indicates that the diet is more prone to promoting inflammation, while a lower DII score indicates that the diet is more resistant to promoting inflammation (12). But there are other health conditions which do not correlate with DII, recently demonstrated in osteoporosis (13).

Thus, exploring the relationships between COPD, diet and inflammation will provide some clues for future prevention or treatment of COPD disease. However, to the best of our knowledge, the correlation between DII and COPD has not yet been studied. In the present study, 9,929 participants were obtained from the 2013 to 2018 National Health and Nutrition Examination Survey (NHANES) to explore the relationship between dietary inflammation and COPD.

## Methods

### Data Source and Study Design

The data were obtained from the 2013 to 2018 Third National Health and Nutrition Examination Survey (NHANES III) (14). NHANES is a cross-sectional investigation that collects health and nutritional information from the non-institutionalized U.S. population. The NHANES website provides detailed information about the study design, interviews, demographics, dietary assessment, physical examination, and laboratory data. The study sample (*n* = 9,926) was limited to adults aged ≥40 years and excluded participants without dietary information or COPD diagnosis history. The participants underwent interviews and medical examinations and completed 24-h (24HR) diet recalls. The participants were categorized based on COPD history and the DII according to interviews and reliable 24HR diet recalls. Serum samples were collected and shipped to the Collaborative Laboratory Services, Ottumwa, Iowa, for the analysis of high-sensitivity C-reactive protein (hs-CRP) by near infrared particle immunoassay rate methodology. Complete blood count was performed on a Coulter DxH 800 analyzer using SP's EDTA blood tubes. Ethical approval was obtained from the CDC/NCHS Ethics Review Board, and informed consent was obtained from all participants.

### Calculation of the DII

The method for calculating the DII was introduced by Shivappa (11) which including three steps. Step1 is to get the Z-score: DII calculations are based on dietary intake data then linked to a regionally representative world database, providing a robust estimated mean and standard deviation for each parameter. These then become the multipliers to express an individual's exposure relative to the “standard global mean” as a *Z*-score. It is calculated by subtracting the global daily mean intake, dividing by its standard deviation, converting the value to a percentile score, doubling each percentile score and subtracting “1” to achieve a symmetrical distribution. Step 2: the centered percentile value for each food parameter was then multiplied by the corresponding “overall food parameter-specific inflammatory effect score” (11) to obtain the “food parameter-specific DII score.” Step 3: by summing each “food parameter-specific DII score,” we can achieve an individual “overall DII score.” Dietary data collected by 24HR diet recall interviews were used to calculate DII scores as described previously (15). In the present study, twenty-seven nutrients were used to calculate the DII score (16): alcohol, vitamin B12/B6, β-carotene, caffeine, carbohydrates, cholesterol, energy, total fat, fiber, folic acid, Fe, Mg, monounsaturated fatty acids (MUFAs), niacin, n-3 fatty acids, protein, polyunsaturated fatty acids (PUFAs), riboflavin, saturated fat, Se, thiamine, vitamin A/C/D/E, and Zn. Additionally, even the numbers of nutrients applied for the calculation of DII is <30, the DII scores are still convincing (16, 17).

### Statistical Analysis

The data were processed by R version 2.1.1 and SPSS version 20.0. Student's *t*-test or one-way ANOVA was performed for the comparison of continuous variables followed by LSD or Dunnett's T3 for *post hoc* multiple comparisons, and the choice of *post hoc* multiple comparisons was based on the Levene statistic in the homogeneity of variance test. The chi-square test was used to compare the constituent ratio of each group. Logistic regression was performed to assess the association between COPD and DII after adjusting for all the covariates. In all analyses, differences were considered statistically significant at a value of *p* < 0.05.

## Results

### Characteristics of Participants

**Figure 1:**
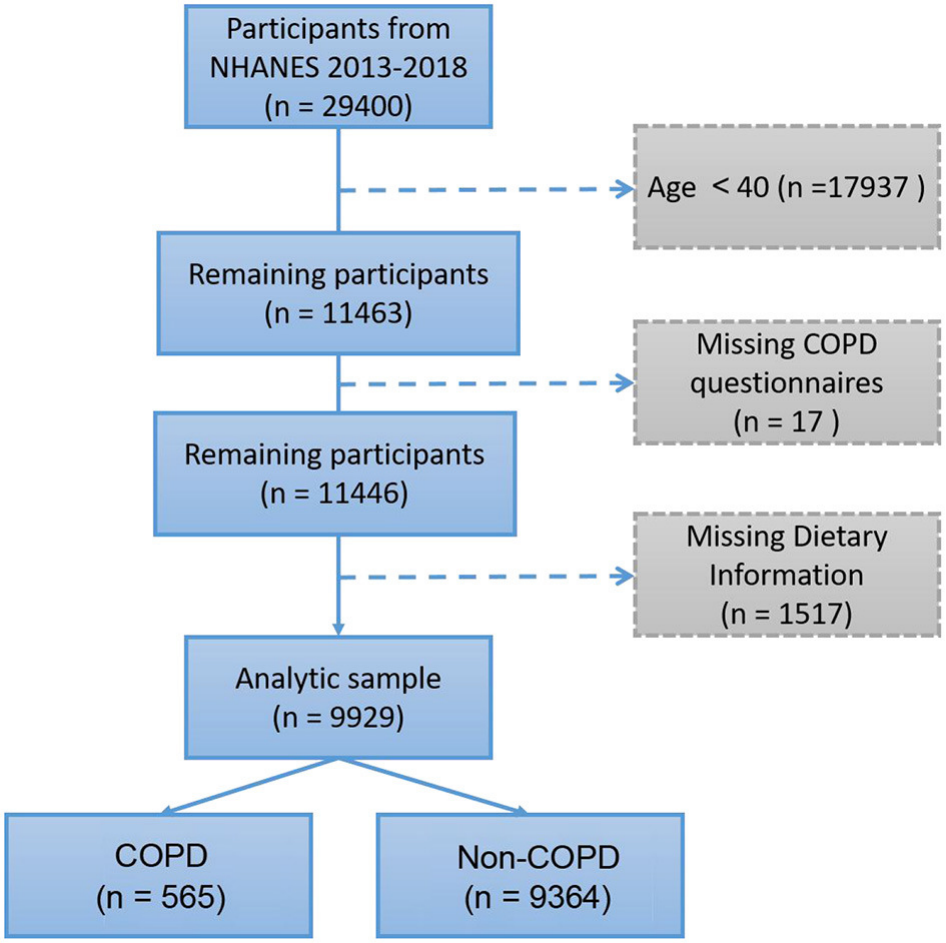
Flow diagram of study participants.

According to the epidemiologic features of COPD, participants under 40 years old were excluded. The present study included a total of 9,929 participants (Figure 1). We identified COPD in 565 (5.69%) participants according to the “Medical Conditions” questionnaire. The demographics and characteristics of the present study participants are described in Table 1. COPD was more prevalent in older males (≥60); men had a higher proportion of COPD than women (53.98 vs. 46.02%). However, body mass index (BMI) showed no significant difference between COPD and non-COPD participants (30.30 ± 8.67 vs. 29.81 ± 6.68, *p* = 0.198). In the COPD group, the proportion of non-Hispanic white individuals was significantly higher than that in the non-COPD group (66.73 vs. 37.97%). In addition, COPD participants had lower education levels and annual family income; more importantly, they had a higher smoking rate (43.72 vs. 15.75%), white blood cell count (WBC) and hs-CRP (*p* < 0.001). In addition, the DII calculated from 27 dietary compositions was higher in COPD participants (0.429 ± 1.809 vs. −0.191 ± 1.791, *p* < 0.001).

**Table 1:** Demographics and characteristics of participants from NHANES 2013–2018.

**Characteristic**	**COPD**	**Non-COPD**	***p***
	**(*N* = 565, 5.69%)**	**(*N* = 9,364, 94.31%)**	
**Age, years**, ***N*** **(%)**			
40–50	42 (7.43%)	2,389 (25.51%)	<0.001^a^
50–60	129 (22.83%)	2,316 (24.73%)	
≥60	394 (66.22%)	4,659 (49.75%)	
Mean ± SD	66.34 ± 10.51	59.45 ± 11.97	<0.001^b^
**Sex**, ***N*** **(%)**			
Male	305 (53.98%)	4,495 (48.00%)	0.005^a^
Female	260 (46.02%)	4,869 (52.00%)	
**BMI, kg/m**^**2**^, ***N*** **(%)**			
<20	44 (7.79%)	286 (3.05%)	<0.001^a^
20–25	109 (19.29%)	1,908 (20.38%)	
25–30	159 (28.14%)	3,183 (33.99%)	
≥30	241 (42.65%)	3,886 (41.50%)	
Mean ± SD	30.30 ± 8.67	29.81± 6.68	0.198^b^
Missing	12 (2.12%)	101 (1.08%)	
**Race**, ***N*** **(%)**			
Mexican American	23 (4.07%)	1,367 (14.60%)	<0.001^a^
Other hispanic	25 (4.42%)	1,040 (11.11%)	
Non-hispanic white	377 (66.73%)	3,555 (37.97%)	
Non-hispanic black	92 (16.28%)	2,080 (22.21%)	
Other race or multiracial	48 (8.50%)	1,322 (14.12%)	
**Education**, ***N*** **(%)**			
< High school	161 (28.50%)	2,108 (22.51%)	<0.001^a^
High school	173 (30.62%)	2,100 (22.43%)	
>High school	231 (40.88%)	5,145 (54.94%)	
Missing	0	11 (0.01%)	
**Annual family income**, ***N*** **(%)**			
<20,000 USD	232 (41.06%)	1,921 (20.51%)	<0.001^a^
≥20,000 USD	309 (54.69%)	6,865 (73.31%)	
Missing	24 (4.25%)	578 (6.17%)	
**Current smoking status**, ***N*** **(%)**			
Smoking	247 (43.72%)	1,475 (15.75%)	<0.001^a^
Non-smoking	240 (42.48%)	2,867 (30.62%)	
Missing	78 (13.81%)	5,022 (53.63%)	
**WBC, 1,000 cell/μL**, **mean ± SD**	8.13 ± 2.36	7.23 ± 4.90	<0.001^b^
**Hs-CRP, mg/L**, **Mean ± SD**	7.75 ± 13.94	4.40 ± 8.49	<0.001^b^
**Dietary inflammatory index**, **mean ± SD**	0.429 ± 1.809	−0.191 ± 1.791	<0.001^b^

*Data are presented as N% (χ^2^ test) and mean ± SD (independent t-test), which are denoted by ^a^ and ^b^, respectively*.

### Characteristics of COPD Participants by DII Tertile

All 9,929 individuals were classified into three groups according to DII tertiles: T1 (DII: −4.996 to −1.092), T2 (DII: −1.089 to 0.612), and T3 (DII: 0.613 to 4.844). The characteristics of the 565 COPD participants according to DII are shown in Table 2: T1 (DII: −3.795 to −1.107) (127 out of 3,309 individuals), T2 (DII: −1.077 to 0.612) (175 out of 3,310 individuals), and T3 (DII: 0.625 to 4.844) (263 out of 3,310 individuals). More individuals (*n* = 263) belonged to the T3 group, indicating that pro-inflammatory food consumption seemed more frequent in COPD patients, while only 127 and 175 individuals belonged to the T1 and T2 groups, respectively. Intriguingly, the elderly COPD participants had a lower DII (T1 vs. T2 vs. T3: 68.21 ± 9.41 vs. 65.90 ± 10.43 vs. 63.58 ± 10.74, *p* < 0.001), and they exhibited significant differences in the *post hoc* test. However, there was no significant difference in WBC or hs-CRP. In addition, individuals from the T1 (28.54 ± 7.14 kg/m^2^) group compared with those from the T2 (31.02 ± 9.13 kg/m^2^) or T3 (30.69 ± 8.95 kg/m^2^) groups were more likely to have a lower BMI (*p* < 0.05), a higher education level (*p* < 0.01), a higher family income (*p* < 0.01), and a lower smoking rate (*p* < 0.01).

**Table 2:** Characteristics of COPD participants aged 40 years and above by dietary inflammatory index (DII) tertiles.

**Characteristic**	**Tertiles of dietary inflammatory index**			***p***
	**T1**	**T2**	**T3**	
	**(−3.795 to −1.107)**	**(−1.077 to 0.612)**	**(0.625 to 4.844)**	
***N***	127	175	263	
**Dietary inflammatory index**,	**–**2.006 ± 0.660	**–**0.210 ± 0.482	2.029 ± 0.983	
**Age, years**, ***N*** **(%)**				
40–50	3 (2.36%)	11 (6.29%)	28 (10.65%)	0.003^a^
50–60	22 (17.32%)	37 (21.14%)	70 (26.62%)	
≥60	102 (80.31%)	127 (72.57%)	165 (62.74%)	
Mean ± SD	68.21 ± 9.41^c^^*^^d^^***^	65.90 ± 10.43^e^^*^	63.58 ± 10.74	<0.001^b^
**Sex**, ***N*** **(%)**				
Male	92 (72.44%)	101 (57.71%)	112 (42.59%)	<0.001^a^
Female	35 (27.56%)	74 (42.29%)	151 (57.41%)	
**BMI, kg/m**^**2**^, ***N*** **(%)**				
<20	13 (10.24%)	12 (6.86%)	19 (7.22%)	0.420^a^
20–25	29 (22.83%)	29 (16.57)	51 (19.39%)	
25–30	40 (31.50%)	50 (28.57%)	69 (26.24%)	
≥30	44 (34.65%)	77 (44.00%)	120 (45.63%)	
Mean ± SD	28.54 ± 7.14^c^^*^^d^^*^	31.02 ± 9.13	30.69 ± 8.95	0.032^b^
Missing	1 (0.79%)	7 (4.00%)	4 (1.52%)	
**Race**, ***N*** **(%)**				
Mexican American	8 (6.30%)	4 (2.29%)	11 (4.18%)	0.152^a^
Other hispanic	5 (3.94%)	11 (6.29%)	9 (3.42%)	
Non-hispanic white	90 (70.87%)	122 (69.71%)	165 (62.74%)	
Non-hispanic black	14 (11.02%)	27 (15.43%)	51 (19.39%)	
Other race or multiracial	10 (7.87%)	11 (6.29%)	27 (10.27%)	
**Education**, ***N*** **(%)**				
< High school	21 (16.54%)	55 (31.43%)	85 (32.32%)	0.001^a^
High school	38 (29.92%)	47 (26.86%)	88 (33.46%)	
>High school	68 (53.54%)	73 (41.71%)	90 (34.22%)	
**Annual family income**, ***N*** **(%)**				
<20,000 USD	38 (29.92%)	70 (40.00%)	124 (47.15%)	0.002^a^
≥20,000 USD	87 (68.50%)	94 (53.71%)	128 (48.67%)	
Missing	2 (1.57%)	11 (6.29%)	11 (4.18%)	
**Current smoking status**, ***N*** **(%)**				
Smoking	43 (33.86%)	69 (38.43%)	135 (51.33%)	0.001^a^
Non-smoking	71 (55.91%)	75 (42.86%)	94 (35.74%)	
Missing	13 (10.24%)	31 (17.71%)	34 (12.93%)	
**WBC, 1,000 cell/μL**	8.01 ± 2.17	7.89 ± 2.22	8.36 ± 2.53	0.114^b^
**Hs-CRP, mg/L**	6.75 ± 15.72	6.70 ± 9.41	8.91 ± 15.42	0.319^b^

*Data are presented as N% (χ^2^ test) and mean ± SD (independent t-test), which are denoted by ^a^ and ^b^, respectively.*

c,d,e *Post hoc analysis between T1 and T2, T1 and T3, and T2 and T3, respectively*.

* *p < 0.05*,

*** *p < 0.001*.

### Association Between COPD and DII

Table 3 represents the crude and adjusted odds ratios for the association between COPD and DII.

**Table 3:** Logistic regression analysis of DII for COPD in participants above 40 years old in NHANES (2013–2018).

	**OR**	**95% CI**	***p***
**Crude model**			
**Tertiles of DII**			<0.001
T1 (−3.795 to −1.107)	0.462	0.372–0.575	<0.001
T2 (−1.077 to 0.612)	0.647	0.531–0.788	<0.001
T3 (0.625 to 4.844)	1.0 (Ref.)	1.0 (Ref.)	
**Adjusted model**			
**Tertiles of DII**			0.011
T1 (−3.795 to −1.107)	0.709	0.512–0.982	0.039
T2 (−1.077 to 0.612)	0.645	0.475–0.877	0.005
T3 (0.625 to 4.844)	1.0 (Ref.)	1.0 (Ref.)	
**Age, years**	1.049	1.035–1.062	<0.001
**Sex**			
Male	0.991	0.758–1.297	0.950
Female	1.0 (Ref.)	1.0 (Ref.)	
**BMI, kg/m** ^**2**^	1.018	0.999–1.037	0.059
**Race**			<0.001
Mexican American	0.196	0.088–0.438	<0.001
Other hispanic	0.540	0.276–1.057	0.072
Non-hispanic white	1.646	1.059–2.557	0.027
Non-hispanic black	0.615	0.368–1.029	0.064
Other race or multiracial	1.0 (Ref.)	1.0 (Ref.)	
**Education**			0.040
< High school	1.0 (Ref.)	1.0 (Ref.)	
High school	0.888	0.629–1.253	0.498
>High school	0.671	0.482–0.934	0.018
**Annual family income**			<0.001
<20,000 USD	1.0 (Ref.)	1.0 (Ref.)	
≥20,000 USD	0.589	0.450–0.772	<0.001
**Current smoking status**			
Smoking	2.690	1.985–3.645	<0.001
Non-smoking	1.0 (Ref.)	1.0 (Ref.)	
**WBC, 1,000 cell/μL**	1.036	0.996–1.078	0.075
**Hs-CRP, mg/L**	1.017	1.007–1.027	0.001

*Ref. means reference group*.

In the crude model, DII as a categorical variable was statistically significantly associated with COPD; those in DII T1 had 0.462-fold lower odds of COPD (OR = 0.462, 95% CI 0.372–0.575, *p* < 0.001) than those in T3. After adjusting for age, sex, BMI, race, education, family income, smoking, WBC, and hs-CRP, the association was slightly weakened. However, in the adjusted model, there was still a significant association between lower DII levels and decreased COPD prevalence (OR = 0.709, 95% CI 0.512–0.982, *p* = 0.039). Besides, in the adjusted model, age, race, education, annual family income, current smoking status, and hs-CRP were significantly correlated with COPD statues.

## Discussion

Using the 2013–2018 NHANES data, we explored the association between COPD and the DII in US adults above 40 years old. We found that COPD participants had significantly higher levels of WBC, hs-CRP, and DII than those without a COPD diagnosis history. Thus, COPD was more prevalent in individuals with higher DII scores, and higher DII scores were related to a higher BMI, lower education, lower family income, higher smoking rate, and certain races, such as non-Hispanic white.

The close relationship between COPD and inflammation has been demonstrated for many years (18). Previous studies have suggested that inflammation increases the risk of acute exacerbation of COPD (AECOPD) (19), while anti-inflammation by glucocorticoids is a common strategy in alleviating COPD (20). Emerging evidence indicates that dietary patterns such as the Mediterranean diet significantly affect levels of inflammation (21). Another investigation indicated that vitamin D, a nutrient partly derived from food supplement, is associated lower risk of obstructive lung disease (22). Therefore, it is plausible that dietary modifications would exert profound impacts on the inflammatory response of COPD. Malnutrition or cachexia is accompanied by advanced COPD (23). Suitable supplementation of nutrients along with a low-inflammation diet might be essential. However, no studies have explored the association between DII and COPD in adults over 40 years old. This investigation provides some information about the relationship between COPD and diet. The DII score was positively correlated with COPD diagnosis. One potential mechanism is that the nutrients that are digested and absorbed into circulation directly affect pulmonary disease. Another interesting finding of this study is that participants with higher age seem to have lower DII scores. These results might be due to the appetites of individuals at different ages (24). Additionally, older individuals have a different gut microbial composition (25, 26), which may affect the metabolism of the host body under stimulation with different diets.

Common pro-inflammatory foods are red meat, refined carbohydrates, sweetened beverages, sweets, fried food, margarine, etc. (Supplementary Table 1) (27, 28). High consumption of processed red meat increases the risk of chronic obstructive pulmonary disease (29, 30). Previous studies have suggested that pro-inflammatory diet is associated with white blood cell counts increase (31), C-reactive protein (CRP) is a biomarker for assessing acute exacerbations of COPD (32). The effect of pro-inflammatory diets on COPD may be through the increase in WBC and hs-CRP levels, but in the current study, we cannot test specific hypotheses. Therefore, future longitudinal studies can consider the potential mechanism of diet-driven inflammation in COPD. Similarly, future research should determine the use of anti-inflammatory diets (for example, increase green leafy vegetables, herbs, spices, and certain fruits and reduce the consumption of sweets, red meat) to reduce WBC and hs-CRP levels, thereby reducing COPD occurrence. Age, race, education, annual family income, current smoking status, and hs-CRP are significantly related with COPD statues in our study. Smoking and age are two most important risk factors of COPD progress (33–35). Besides, education, annual family income and race are strong social determinants of health status in COPD patients (36–38).

The major limitations of our study is our DII is not compared with the energy-adjusted DII (E-DII), which construct a referent database of energy-adjusted nutrient scores on the basis of data from the same 11 countries used to compute the DII, with 16 publications so far (39). It would be very import to compare different types of DII score across studies conducted virtually anywhere in the world, meanwhile, the E-DII cannot be computed without access to the unique comparative databases, so we failed to perform the comparison in our study (39). Hebert J et al. reported that flavonoids as systemically important regulators of combustion should be included in the DII score, but flavonoids were not included in this study (39). It's also worth noting that we did not have enough people aged 40–50 in this study. However, the proportion of COPD patients in the 40–50 year old group was lower than that in the 50–60 and ≥60 year old groups, which is consistent with the previous studies, for that COPD is a kind of aging disease, and the incidence of disease gradually increases with age (35). Besides, age is not the most important observation indicator in this study, we think our results might still worthy of attention.

## Conclusion

There is a positive association between the DII and COPD in adults over 40 years old, and the DII might be clinically used as a predictor for the risk of COPD. However, the potential mechanism by which food drives inflammation in COPD needs further investigation.

## Data Availability Statement

Publicly available datasets were analyzed in this study. This data can be found here: https://wwwn.cdc.gov/nchs/nhanes/Default.aspx.

## Author Contributions

HL designed the research. ZL analyzed the data. XT wrote the paper. XM, YZ, BZ, GZ, and YH revised and improved the grammar and data interpretation. LF and GH revised the paper. All authors read and approved the final manuscript.

## Funding

We sincerely acknowledge the financial support of the National Natural Science Foundation of China (81772287, 81371902), the Natural Science Foundation of Fujian Province (2020J011241, 2021J05283, 2021J05287), and the Guangdong Basic and Applied Basic Research Foundation (2019A1515111063).

## Conflict of Interest

The authors declare that the research was conducted in the absence of any commercial or financial relationships that could be construed as a potential conflict of interest.

## Publisher's Note

All claims expressed in this article are solely those of the authors and do not necessarily represent those of their affiliated organizations, or those of the publisher, the editors and the reviewers. Any product that may be evaluated in this article, or claim that may be made by its manufacturer, is not guaranteed or endorsed by the publisher.
