# Supplementary material for: Association Between Diet-Related Inflammation and COPD: Findings From NHANES III
Source: Front Nutr. 2021 Oct 18;8:732099. doi: 10.3389/fnut.2021.732099 (PMC8558221; doi:10.3389/fnut.2021.732099)
Supplement: Supplementary file 1 [file Table_1.DOCX]

Table S1 Common pro-inflammatory foods

| **Food names** | **Reference** |
| --- | --- |
| red meat | ([1](#_ENREF_1)) |
| processed meat |  |
| organ meat |  |
| refined carbohydrates |  |
| sweetened beverages |  |
| sweets | ([2](#_ENREF_2)) |
| processed food |  |
| high fat meat |  |
| fried food |  |
| salt |  |
| margarine |  |
| snacks |  |
| ice cream |  |
| mayonnaise |  |
| stuffed biscuits | ([3](#_ENREF_3)) |
| fried snacks |  |
| Artificial food dyes | ([4](#_ENREF_4)) |
| carbonated drinks | ([5](#_ENREF_5)) |
| "fast" foods | ([6](#_ENREF_6)) |

1. J. Li, D. H. Lee, J. Hu, F. K. Tabung, Y. Li, S. N. Bhupathiraju, E. B. Rimm, K. M. Rexrode, J. E. Manson, W. C. Willett, E. L. Giovannucci and F. B. Hu: Dietary Inflammatory Potential and Risk of Cardiovascular Disease Among Men and Women in the U.S. *J Am Coll Cardiol*. (2020) 76: 2181-2193. doi:10.1016/j.jacc.2020.09.535

2. C. M. Preda, T. Manuc, A. Chifulescu, D. Istratescu, E. Louis, C. Baicus, I. Sandra, M. M. Diculescu, C. Reenaers, C. van Kemseke, M. Nitescu, C. Tieranu, M. Popescu, L. Tugui, A. Andrei, C. A. Ciora, I. S. Gherorghe and M. Manuc: Diet as an environmental trigger in inflammatory bowel disease: a retrospective comparative study in two European cohorts. *Rev Esp Enferm Dig*. (2020) 112: 440-447. doi:10.17235/reed.2020.6552/2019

3. F. Gomes de Luna Mde, J. R. Gomes de Luna, G. B. Fisher, P. C. de Almeida, D. Chiesa and M. G. Carlos da Silva: Factors associated with asthma in adolescents in the city of Fortaleza, Brazil. *J Asthma*. (2015) 52: 485-91. doi:10.3109/02770903.2014.984841

4. A. Vojdani and C. Vojdani: Immune reactivity to food coloring. *Altern Ther Health Med*. (2015) 21 Suppl 1: 52-62.

5. A. Fahim, M. S. Ilyas, F. H. Jafari and F. Farzana: Effect of carbonated drinks on wound healing of oral epithelium. *J Oral Biol Craniofac Res*. (2016) 6: 49-53. doi:10.1016/j.jobcr.2015.08.005

6. L. G. Wood: Diet, Obesity, and Asthma. *Ann Am Thorac Soc*. (2017) 14: S332-S338. doi:10.1513/AnnalsATS.201702-124AW
